# Mechanisms and Alterations of Cardiac Ion Channels Leading to Disease: Role of Ankyrin-B in Cardiac Function

**DOI:** 10.3390/biom10020211

**Published:** 2020-01-31

**Authors:** Holly C. Sucharski, Emma K. Dudley, Caullin B.R. Keith, Mona El Refaey, Sara N. Koenig, Peter J. Mohler

**Affiliations:** 1 Dorothy M. Davis Heart and Lung Research Institute, The Ohio State University Wexner Medical Center, Columbus, OH 43210, USA; holly.sucharski@osumc.edu (H.C.S.); emma.dudley@osumc.edu (E.K.D.); Mona.elrefaey@osumc.edu (M.E.R.); peter.mohler@osumc.edu (P.J.M.); 2 Departments of Physiology and Cell Biology and Internal Medicine, Division of Cardiovascular Medicine, The Ohio State University College of Medicine and Wexner Medical Center, Columbus, OH 43210, USA

**Keywords:** ankyrin-B, *ANK2*, ion channels, cardiovascular disease

## Abstract

Ankyrin-B (encoded by *ANK2*), originally identified as a key cytoskeletal-associated protein in the brain, is highly expressed in the heart and plays critical roles in cardiac physiology and cell biology. In the heart, ankyrin-B plays key roles in the targeting and localization of key ion channels and transporters, structural proteins, and signaling molecules. The role of ankyrin-B in normal cardiac function is illustrated in animal models lacking ankyrin-B expression, which display significant electrical and structural phenotypes and life-threatening arrhythmias. Further, ankyrin-B dysfunction has been associated with cardiac phenotypes in humans (now referred to as “ankyrin-B syndrome”) including sinus node dysfunction, heart rate variability, atrial fibrillation, conduction block, arrhythmogenic cardiomyopathy, structural remodeling, and sudden cardiac death. Here, we review the diverse roles of ankyrin-B in the vertebrate heart with a significant focus on ankyrin-B-linked cell- and molecular-pathways and disease.

## 1. Introduction: Ankyrin Proteins

The ankyrin family of polypeptides was first identified in the erythrocyte plasma membrane in 1979 by Bennett and Stenbuck [1]. Following this discovery, ankyrin was discovered in various organs and cell types, including brain [2,3,4] and myogenic cells [5,6]. We now know that ankyrins are derived from three ankyrin genes: *ANK1, ANK2,* and *ANK3* [7,8,9]. *ANK1* encodes ankyrin-R (AnkR), *ANK2* encodes ankyrin-B (AnkB), and *ANK3* encodes ankyrin-G (AnkG) (Table 1).

Ankyrin-R is primarily expressed in erythrocytes and was the first ankyrin identified as an adaptor protein [1], linking erythrocyte membrane proteins to the actin-based cytoskeleton [30]. AnkR is important for the structural integrity and organization of the erythrocyte membrane. Moreover, loss-of-function variants in human *ANK1* have been linked to ~50% of hereditary spherocytosis cases [31], a complex form of hemolytic anemia that affects 1:2000 people of Northern European descent [26] and results from a loss of membrane surface tension in red blood cells [32]. AnkR regulates erythrocyte membrane expression of CD44 [15], Na^+^/K^+^ ATPase (NKA) [16], and the Rh type A glycoprotein [17]. Although AnkR is primarily expressed in erythrocytes, AnkR is also expressed in myelinated axons [10]. *ANK1* also encodes four small ankyrin-1 isoforms (sAnk1.5, 1.6, 1.7, and 1.9) that are highly expressed in striated muscle. These isoforms bind obscurin and stabilize the sarcoplasmic reticulum in striated muscle [11].

Ankyrin-B was first identified in the brain [8] and has since been identified as a critical adaptor and scaffolding protein in the heart, that mediates the interaction of integral membrane proteins with the spectrin-actin cytoskeletal network [13,33]. *ANK2* encodes multiple isoforms that may contribute to disease. AnkB will be reviewed in detail below.

Ankyrin-G plays an important role across multiple excitable tissues. In the brain, AnkG links integral membrane proteins with the actin/spectrin-based membrane skeleton at axon initial segments (AIS) including Na_V_1.6, βIV spectrin, and L1CAMs [21,22,33]. In the heart, AnkG is required for localization of Na_V_1.5 and CaMKII to the cardiomyocyte intercalated disc [13,14,34]. In mice selectively lacking AnkG expression in cardiomyocytes, βIV-spectrin and Na_V_1.5 expression and localization are disrupted, and voltage-gated Na_V_ channel activity (I_Na_) is significantly decreased. These animals experience a reduction in heart rate, impaired atrioventricular conduction, increased PR intervals, and increased QRS intervals [14]. Further, AnkG cKO mice display arrhythmias in response to adrenergic stimulation. In humans, an *SCN5A* variant in the AnkG-binding motif of Na_V_1.5 has been associated with Brugada syndrome and arrhythmia [12]. This same variant is a loss-of-function variant when expressed in primary cardiomyocytes. Similar to other ankyrin genes, *ANK3* encodes multiple isoforms of AnkG. Giant AnkG is a 480-kD protein required for proper AIS and node of Ranvier assembly due to the clustering of Na_V_ channels [35]. Human variants affecting 480-kD AnkG are associated with severe cognitive disability [29]. The role of Giant AnkG isoforms in the heart is currently unknown and is an important area for future research.

AnkB and AnkG are ubiquitously expressed, but their functions are distinct. Although AnkG plays a crucial role in the brain, variants in AnkG have been connected to Brugada syndrome [12] and, more recently, dilated cardiomyopathy [28]. Although AnkB and AnkG have similar structures, AnkG partners with proteins at the intercalated disc, including plakophilin-2 [23] and Na_V_1.5 [14], while AnkB is crucial for the expression and localization of ion channels at the sarcoplasmic reticulum, transverse-tubules, and plasma membrane [12]. However, Roberts et al. recently identified small populations of AnkB at the intercalated disc [27]. Cardiomyocytes from mice heterozygous for a null mutation in ankyrin-B display mislocalization and a decrease in expression of Na^+^/Ca^2+^ exchanger (NCX) and Na^+^/K^+^-ATPase (NKA) [36]. Further, Roberts et al. demonstrated that β-catenin is a novel AnkB-binding partner, where β-catenin localization is disrupted in individuals with *ANK2* variants who presented with arrhythmogenic right ventricular cardiomyopathy (ARVC) [27]. Importantly, ankyrins -G and -B retain non-overlapping, non-compensatory functions despite their similarity in sequence. Distinct from AnkG-associated disease, variants in AnkB are tied to a specific set of clinical phenotypes, including susceptibilities to sinus node dysfunction and acquired heart diseases such as atrial fibrillation [12] and heart failure [37]. Ankyrin specificity, at least in part, is attributed to an autoinhibitory linker peptide between the membrane-binding domain (MBD) and spectrin-binding domain (SBD), which prevents AnkB from binding with protein partners [38]. Further specificity is attributed to key roles of the divergent C-terminal domains of AnkB and AnkG. Additional mechanisms underlying ankyrin specificity in vivo are a key area for future research.

## 2. Ankyrin-B Isoforms

Similar to *ANK1* and *ANK3*, *ANK2* generates multiple gene products. The diversity of known and potential gene products is large and includes splice products defined as small, canonical, and Giant AnkB isoforms [13]. In fact, The National Center for Biotechnology Information gene database (NCBI Gene) lists 49 transcript variants that match the known RefSeq (NM) and 20 transcript variants that match the model RefSeq (XM), yet most of these transcript variants have not been identified in tissue or cells.

AnkB-188 and AnkB-212 are two ankyrin-B isoforms present in the heart [24]. AnkB-188 is expressed in human ventricular cardiomyocytes and regulates NCX expression, whereas AnkB-212 is expressed in cardiomyocytes and skeletal muscle, is localized to the M-line, and exclusively interacts with obscurin [24]. Furthermore, 440kD AnkB (Giant AnkB) is the result of the insertion of a 6.4 kb exon between the SBD and death domain (DD). To date, expression of Giant AnkB has only been studied in neurons and was recently associated with autism [25]. Giant AnkB regulates the expression and localization of L1CAMs in neurons and predominates in unmyelinated axons to control axonal branching. A mouse model deficient in Giant AnkB shows increased axonal branching and a transient increase in excitatory synapses during postnatal development [39]. Although there are a host of ankyrin-B isoforms in the heart, the primary isoform is canonical 220-kD AnkB.

Canonical AnkB is an adaptor protein that acts as a pivotal regulator in the localization and organization of ion channels, structural proteins, signaling molecules, and adaptor proteins [40,41]. Deficiency in AnkB results in mislocalization and altered expression of multiple membrane and cytoskeletal proteins, including NCX [12], NKA [18], ATP-sensitive inward rectifier K^+^ channel (Kir6.2) [13], voltage-gated calcium channel (Ca_V_1.3) [19], and βII-spectrin [20], causing ion imbalance and dysregulation of cellular signaling [18].

Animal models have been fundamental in illustrating the role of AnkB in the heart. AnkB^+/−^ myocytes display reduced expression and improper localization of AnkB-binding partners, as well as strong electrical phenotypes including extrasystoles, early afterdepolarizations (EADs), and delayed afterdepolarizations (DADs) following adrenergic stimulation. The mechanism underlying AnkB-associated arrhythmias is attributed to improper calcium handling via disrupted organization of AnkB-binding partners [36]. Through the use of a canine cardiomyocyte model, Chu et al. predicted that mislocalization of the NKA and its subsequent uncoupling from the NCX in AnkB^+/−^ cardiomyocytes disturbs Ca^2+^ and Na^+^ currents to predispose the cell to action potential prolongation [42].

## 3. Ankyrin-B Structure and Binding Partners

**Figure 1:**
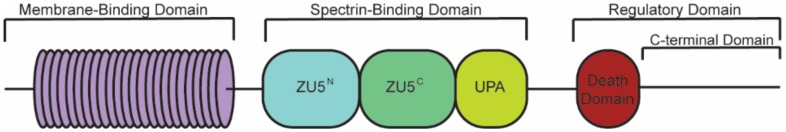
Structure of canonical ankyrin-B. Canonical ankyrin proteins share four domains: a membrane-binding domain (MBD), spectrin-binding domain (SBD), death domain (DD), and C-terminal domain (CTD). The MBD consists of 24 *ANK* repeats that are defined by their secondary structure and aid in protein folding regulation. The SBD consists of ZU5N, ZU5C, and UPA domains that are important for binding βII-spectrin and supporting cardiomyocyte structure. The DD and CTD comprise the regulatory domain.

**Figure 2:**
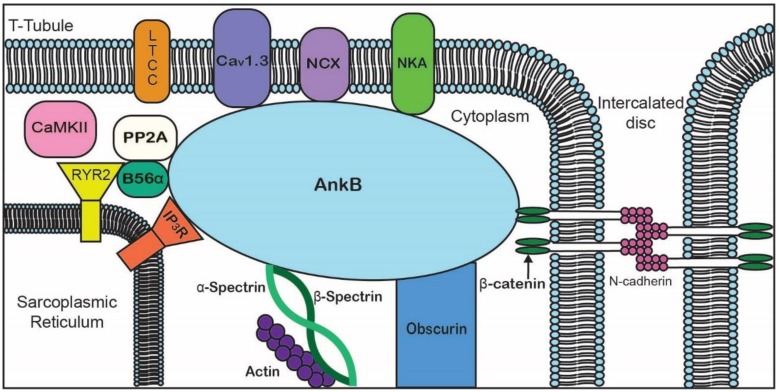
Representative diagram of ankyrin-B-binding partners to emphasize the importance of AnkB in the localization of ion channels, transporters, pumps, and structural proteins for proper cardiomyocyte function.

Canonical ankyrin proteins share a similar structure composed of an MBD, SBD, and a regulatory domain (RD) that is comprised of a death domain (DD) and C-terminal domain (CTD) (Figure 1). The MBD of AnkB is divided into four subdomains composed of 24 *ANK* repeats, which are defined by their repeated alpha-helical structure [43]. *ANK* repeats are not specific to the three canonical ankyrin proteins and are present across a range of functionally diverse proteins including SHANK, BARD1, and ANKRD. The AnkB MBD regulates the localization of ion channels and transporters (Figure 2 and Table 2).

The SBD is a highly conserved region of most ankyrin proteins, where the AnkB SBD is composed of a ZU5^N^-ZU5^C^-UPA tandem structure. The ZU5^N^ domain binds directly with the spectrin cytoskeleton to aid in the structural integrity of the myocyte [44]. Critical to calcium handling in the heart, the ankyrin ZU5^C^ region of the AnkB-SBD associates with the B56α subunit of protein phosphatase 2A (PP2A), an interaction that localizes PP2A with a primary target, RyR2 [12,13]. The *ANK2* p.Q1283H variant within ZU5c was recently associated with arrhythmia, potentially due to loss of PP2A activity and altered phosphorylation of RyR2 [45].

The AnkB DD, composed of six helices that resemble the death domain of apoptotic-related proteins, is proposed to play a role in the auto-inhibitory functions of AnkB through the binding of the DD to the UPA region of SBD [44]. Additionally, a yeast two-hybrid screen and subsequent co-immunoprecipitation revealed that the AnkB DD binds RAB GTPase activating protein-1 like (RABGAP1L), which is involved in intracellular membrane trafficking within many different cell types, including heart tissue [46]. Although the regulatory domain is the least conserved domain of AnkB, the majority of disease-associated variants have been identified in this region. The DD variant, V1516D, was identified in four individuals with various heart conditions such as atrial fibrillation, drug-induced long QT syndrome (LQTS), exercise-induced ventricular tachycardia, bradycardia, syncope, or a combination of phenotypes [12].

Finally, the AnkB CTD is characterized by its elongated structure and its high composition of charged amino acids [12]. It is highly divergent and interacts with obscurin [47]. The CTD structure allows for inter-domain interactions that are crucial to isoform-specific functions. Intramolecular interactions between the AnkB CTD and MBD are proposed to regulate protein binding interactions. Ankyrin auto-regulatory activity was originally identified in AnkR and its splice variant AnkR 2.2 [48,49]. Within AnkB, yeast two-hybrid studies identified essential amino acid sequences of the CTD and MBD that are required for binding; Arg 37 and Arg 40 of the *ANK* repeat 1 within the MBD and the three amino acid sequence EED within the CTD are shown to facilitate inter-domain binding [12]. Abolishment of this interaction inhibits AnkB-specific targeting of inositol trisphosphate receptor (IP3R) to the sarcoplasmic reticulum, therefore demonstrating that inter-domain interactions are responsible for ankyrin function through regulation of proper protein interactions. Interestingly, however, these amino acid sequences are not required for binding of AnkB MBD with the IP3R, raising questions about the role of the CTD in localization and expression of AnkB-binding partners within the heart.

## 4. Ankyrin-B Variants in Cardiovascular Disease

AnkB variants have been identified in all four domains of the AnkB protein and are linked to a spectrum of cardiovascular phenotypes. AnkB is classically associated with human arrhythmia syndromes, many of which demonstrate incomplete penetrance and variable expressivity [12,50,51,52]. In fact, it is likely that secondary genetic, lifestyle, and/or environmental factors are necessary to cause disease. Ankyrin-B syndrome, originally classified as long QT syndrome type 4, is a heritable arrhythmogenic disease that is the result of loss-of-function mutations in *ANK2*. A p.E1425G variant was discovered in a French family suffering from sinus bradycardia, atrial fibrillation, and sudden cardiac death and was the first to be implicated in AnkB syndrome [12,53].

Although the p.E1425G variant is localized to the AnkB regulatory domain, loss-of-function variants in all four ankyrin domains are now associated with AnkB syndrome [12,54]. Notably, these variants show a range of clinical severity and phenotypes, including torsades de pointes (TdP), ventricular tachycardia, and long QT syndrome, a variability that is reflected at the cellular level [12]. This inconsistency of phenotype puts into the question the mechanics behind AnkB-associated diseases that have yet to be fully elucidated. Although the AnkB MBD is imperative for AnkB interactions and function within the heart, the first disease-associated loss-of-function variant in this domain, p.S646F, was only recently identified within the First Nations of Northern British Columbia [54]. Individuals with this variant also exhibited congenital heart defects, Wolff–Parkinson–White syndrome, and cardiomyopathy. These compounded symptoms are the result of improper NCX localization, subsequent Ca^2+^ overload, and possible disruption of pacemaking activity [12,36].

Variants within the SBD pose distinct mechanisms of disease. Several AnkB variants including the p.R990Q [20], p.A1000P, and p.DAR976AAA are present within the highly conserved ZU5^N^ region of the SBD that directly binds βII-spectrin, disrupting this interaction. Importantly, AnkB co-immunoprecipitates with a larger complex composed of βII-spectrin, NKA, and NCX [20]. Although p.A1000P and p.DAR976AAA demonstrated normal AnkB activity with altered βII-spectrin binding, the p.R990Q variant showed altered AnkB functionality, including an inability to rescue NCX localization in AnkB knockout myocytes and severe arrhythmia attributed to disruption of the AnkB-spectrin interaction [20]. Notably, the p.Q1283H variant within the ZU5^C^ region disrupts PP2A activity via loss of B56α targeting, which increases RyR2 phosphorylation and disrupts the calcium dynamics associated with excitation-contraction (EC) coupling [45]. Notably, alterations in RyR2 activity are associated with dilated cardiomyopathy and offer an additional area for future investigation.

Variants in *ANK2* have also been associated with sinus node disease (SND) [12] and most recently with arrhythmogenic cardiomyopathy (ACM) [27]. In fact, the p.E1425G variant segregated with sinus node disease with nearly complete penetrance [45]. Similar to *ANK2* loss of function mutations, *ANK2* transection in chromosome 4, leading to *ANK2* haploinsufficiency, was associated with ankyrin-B syndrome [55].

Although AnkB is commonly associated with arrhythmias, variants in *ANK2* have also been associated with structural heart disease. Lopes et al. found that individuals with *ANK2* variants had a greater maximum wall thickness in the left ventricle, in hypertrophic cardiomyopathy [50]. AnkB has also been linked to acquired heart disease and tissue remodeling following infarct in canine animal models [12]. Following coronary artery occlusion, AnkB mRNA and protein levels decrease in the cardiomyocytes found at the infarct border zone (BZ), as do common AnkB-binding partners including NCX and NKA. These findings provide new insight into the role of AnkB in heart failure and possibly for the cardiac remodeling that supports the creation of arrhythmogenic substrates at the border zone [37].

Recently, *ANK2* variants have been identified in individuals with arrhythmogenic right ventricular cardiomyopathy (ARVC) [27], a disease characterized by a severe structural and electrical cardiac phenotype that involves the fibrofatty replacement of healthy myocardium, malignant arrhythmias, and even sudden cardiac death [56]. The AnkB-p.Glu1458Gly variant was linked to AnkB syndrome but was also identified in a family with AnkB syndrome found to have ARVC at autopsy [27]. A larger screen that encompassed AnkB variants in ACM revealed a loss-of-function variant, AnkB-p.Met1988Thr, which segregated with ARVC-affected family members, where staining of the ventricle tissue showed reduced levels of NCX at the plasma membrane and abnormal Z-line targeting [27]. In order to model the ACM phenotype seen in these patients with loss-of-function AnkB variants and ACM, a cardiomyocyte-specific AnkB knockout mouse was generated (as AnkB null mice die shortly after birth), which developed a phenotype similar to that of human ACM, including dramatic structural abnormalities, biventricular dilation, reduced ejection fraction, cardiac fibrosis, premature death, and exercise-induced death [27]. Although the desmosome was preserved in these mice, β-catenin localization was altered, and β-catenin was demonstrated as a binding partner for the AnkB MBD. Interestingly, when these mice were treated with GSK-3β-inhibitor, a pharmacological activator of β-catenin, it prevented and partially reversed the ARVC phenotype found in these mice [27], providing a hopeful outlook on developing new therapies for patients with AnkB variant-driven ARVC.

## 5. Future Implications

*ANK2* variants are associated with cardiovascular phenotypes including sinus node disease, atrial fibrillation, heart rate variability, catecholaminergic polymorphic ventricular tachycardia (CPVT), ACM, cardiomyopathy, syncope, and sudden cardiac death. We strongly predict that disease penetrance and severity will ultimately be predicated by the interaction between genetic (known as well as currently unknown variants, deletions, etc.) and environmental factors. A key future area of research is to understand the impact of secondary variants and acquired/environmental factors (e.g., ischemia, catecholamines) on ankyrin-B stability and disease.

Despite their variability, the majority of treatment options for AnkB-associated conditions continue to mitigate the clinical symptoms of disease as opposed to their molecular causes. Currently, SND, ARVC, and AnkB syndrome necessitate symptom-dependent approaches ranging from β-blocker administration to pacemaker implantation [12,37,56]. Only recently was SB-216763, a GSK-3β inhibitor, identified to both prevent and reverse ARVC in mice by targeting a specific interaction between AnkB and β-catenin through the Wnt signaling pathway [27], but this therapeutic strategy has not yet been tested. Although the authors posit that the drug may function by altering β-catenin phosphorylation levels, the exact mechanics of its action remain unclear, which highlights the need for future examination. Furthermore, these findings address the structural phenotype of ARVC that is largely absent in SND and AnkB syndrome, again establishing the need for more disease-specific therapeutic options.

The clinical manifestations of AnkB-attributed pathologies are also intriguingly patient specific. For example, cases of ARVC and AnkB syndrome, which comprise disparate structural and electrical phenotypes, have both been linked to the AnkB E1425G variant [27]. This genetic overlap demonstrates that individual AnkB variants are not likely the sole determinants of a given clinical phenotype, but rather are compounded by environmental factors that work in conjunction with additional genetic factors to produce disease variability. Further study is necessary to elucidate the synergistic interactions and intricate molecular pathways that could act as novel therapeutic targets in AnkB-associated illness.

In summary, AnkB is critical for the expression, targeting, and regulation of multiple proteins involved in cardiac excitability, structure, and signaling. AnkB is a pivotal regulator of ion channels and transporters (e.g., NCX and NKA), structural proteins (e.g., βII-spectrin and β-catenin), and calcium regulatory proteins (e.g., RyR2 and PP2A) (Figure 2). Together, the diverse roles of AnkB contribute to EC coupling, cytoskeletal integrity, and signaling pathways required for proper cardiomyocyte function. Variants in AnkB are associated with arrhythmogenic diseases that include both structural and electrical dysfunction. Although AnkB isoforms have been shown to have unique functions in the heart (AnkB-188 and AnkB-212), it is not known if variants in these isoforms contribute to disease. Since AnkB-188 plays a role in localization of NCX and AnkB-212 binds obscurin, variants may lead to M-line disruption in cardiomyocyte conduction and contraction, as knockdown of either isoform resulted in arrhythmic contraction in vitro [24]. Although Giant AnkB has only been studied in the brain, where variants may increase susceptibility to cognitive disorders and autism, its expression and role in heart disease has yet to be determined. Understanding the role of AnkB variants in disease, along with other variants that contribute to the phenotypes described here, is crucial to developing new treatment strategies for complex human disease.

## Figures and Tables

**Table 1:** Brief summary of ankyrin family proteins: ankyrin-R, ankyrin-B, ankyrin-G.

	Ankyrin-R	Ankyrin-B	Ankyrin-G
**Tissue Expression**	erythrocytes [1], myelinated axons [10], striated muscle [11]	ubiquitously expressed, cardiomyocytes (T-tubules, SR, plasma membrane) [12], neurons [8]	ubiquitously expressed, neurons (AIS, and nodes of Ranvier) [13], cardiomyocytes (intercalated disc) [14]
**Examples of Binding Partners**	CD44 [15], NKA [16], Rh type A glycoprotein [17], obscurin [11]	PP2A [12,13], NCX [12], NKA [18], Kir6.2 [12,13], Ca_V_1.3 [19], βII-spectrin [20]	Na_V_1.6, βIV-spectrin, L1CAMs [1,21,22], plakophilin-2 [23] Na_V_1.5 [14]
**Isoforms**	sAnk1.5, 1.6, 1.7, and 1.9 [11]	AnkB-188 and AnkB-212 [24]. Giant AnkB (440-kD)	Giant AnkG (480-kD) [25]
**Disease associated with variants**	hereditary spherocytosis [26]	Ankyrin B syndrome: SCD, SND, AF, LQTS, VT, bradycardia, syncope [12], ARVC [27]	Brugada syndrome [12], dilated cardiomyopathy [28], cognitive disabilities [29]

SR = sarcoplasmic reticulum, AIS = axon initial segments, NKA = Na^+^/K^+^ ATPase, PP2A = protein phosphatase 2A, NCX = Na^+^/Ca^2+^ exchanger, Kir6.2 = inward rectifier potassium channel, Ca_V_1.3 = voltage-gated calcium channel, L1CAMs = L1 family of neural cell adhesion molecules, SCD = sudden cardiac death, SND = sinus node disease, AF = atrial fibrillation, LQTS = long QT syndrome, VT = ventricular tachycardia, ARVC = arrhythmogenic right ventricular cardiomyopathy.

**Table 2:** Ankyrin-B-binding partners in the heart.

Membrane-Binding Domain		Spectrin-Binding Domain	Regulatory Domain
**Ion channels**	**Transporters/Pumps**	β-spectrin	HSP40
IP3R	Anion Exchanger	PP2A	Obscurin
Ca_v_1.3	Na/Ca Exchanger		Ankyrin MBD
Kir6.2	Na/K ATPase		
**Structural**	**Cell adhesion**		
Tubulin β-catenin	L1CAMs		
	β-dystroglycan		
	Dystrophin		

IP3R = 1,4,5 inositol trisphosphate receptor, Ca_V_1.3 = voltage-gated Ca^2+^ channel, Kir6.2 = ATP-sensitive inward rectifier K^+^ channel, PP2A = protein phosphatase 2A, HSP40 = heat shock protein 40, MBD = membrane binding domain, L1CAMs = L1 family of neural cell adhesion molecules.
